# The colour of sauropod skin. A comment on: ‘Fossilized melanosomes reveal colour patterning of a sauropod dinosaur’, (2025) by Gallagher *et al.*

**DOI:** 10.1098/rsos.260429

**Published:** 2026-09-09

**Authors:** Valentina Rossi, Maria E. McNamara

**Affiliations:** School of Biological, Earth and Environmental Sciences, University College Cork, Cork, County Cork, Ireland

**Keywords:** sauropod dinosaurs, colour, integument, soft tissues, melanosomes

Gallagher *et al.* [1] report preserved melanosomes in the integument of juvenile diplodocids from the Late Jurassic of Mother's Day Quarry (Montana, USA). The authors claim that the fossil skin contains melanosomes with two distinct morphotypes and geometries similar to melanosomes associated with iridescent feather colours of extant birds. Based on this evidence, the authors conclude that this study is the first to constrain the colour pattern of sauropod skin. We agree with the authors that some of the microbodies observed in the samples are consistent with melanosomes, the first reported for sauropod soft tissues. We have, however, major concerns regarding the nature of the samples, the measurement of melanosome geometry and the analyses of the geometry data. Additional issues relate to the interpretation of skin layers and chemical analysis. The published dataset and interpretation contain several errors. We, therefore, consider that the conclusions are unsupported by the data.

The first major issue relates to the absence of basic data on the provenance and nature of the samples analysed. The text reports that five specimens were sampled, but no data are provided on the nature of these specimens and on the provenance of the samples. More specifically, it is not clear whether the specimens are partial or complete skeletons or whether they represent isolated soft tissues; the sampling location on the body is not provided. Further, the interpretation that the material analysed represents sauropod tissues is not supported by adequate evidence. The material imaged in figure 1A–D is reported to show polygonal scales, but said scales are not evident. The rock sample pictured in figure 1H–I is identified as showing well-preserved polygonal and rectangular scales. The geometry and size distribution of the component structures, however, are not consistent with sauropod scalation, but rather features formed due to dehydrational contraction, as commonly seen in geological materials during diagenesis. The definition of the purported scalation by the apparent absence of organic material is not consistent with the mode of preservation of the proposed scales in figure 1A–D, which seem to be defined by a dark organic margin.

The second major issue relates to the measurement of melanosome geometry. Gallagher *et al.* [1] measured melanosomes that are embedded in one of two materials: a matrix inferred to be carbonaceous in composition (e.g. figure 2D) and a second matrix inferred to comprise clay minerals (e.g. figure 2C). In each case, the melanosomes may be only partially exposed, and thus should not be used for measurement of melanosome geometry, as it will result in underestimation of the melanosome true long and short axes. For instance, the feature labelled as a disc-shaped melanosome in figure 2E is clearly a melanosome that has been fractured and exposed (possibly during sampling), and that exposed surface is unlikely to be an accurate representation of the geometry of that organelle. The same issue is quite obvious in figure 4C, where the disc-shaped melanosomes could be spherical or ovoid melanosomes that are partially embedded in the carbonaceous matrix. Finally, the scanning electron microscope (SEM) micrographs of melanosomes and their moulds show clear variation in tone consistent with curved surfaces and not discs.

Some melanosomes are preserved as moulds, but these are unexpectedly small in size (e.g. figure 1E) relative to melanosomes described in most extant and fossil vertebrates [2]. Again, this may reflect only partial exposure of the moulds.

The third major issue is that the number of melanosomes measured is extremely low. The authors acknowledge this, and we agree that it can be challenging to identify melanosomes in fossilized soft tissues. That said, a dataset of 16 or 32 melanosomes per sample is insufficient for rigorous analysis of melanosome geometry: the norm is to use at least 50 melanosomes per soft tissue sample. Related to this, we have concerns regarding the analysis and interpretation of the geometry data. The authors have visualized the data using a scatter plot (figure 5). Problematically, rather than plotting measurement data for each melanosome, the authors have plotted only the mean values per sample, which does not allow assessment of the variation of the data. Scatter plots have important limitations for clearly showcasing differences and similarities in melanosome morphologies, and recent studies [2–4] use box plots to better visualize variation and support interpretations.

Critically, major datasets of integumentary melanosome geometry are not included in the analysis by Gallagher *et al.* [1] (e.g. [2,3,5,6]). The assignment of the fossil melanosomes to a particular colour category is therefore not based on current data.

Fourthly, the authors’ application of geometry data from feather melanosomes to infer the colour of the diplodocid integument (i.e. brown; an interpretation that is applied in the art published with the press release) is fundamentally incorrect. Although various studies have demonstrated an association between melanosome geometry and feather colour (e.g. [5,7,8]), previous work [4] has demonstrated that melanosome geometry is not related to skin colour. Even if such a relationship existed, inferences of colour from melanosome geometry data are normally achieved via statistical analysis (e.g. quadratic discriminant analyses; [5]) and not via a simple length–width scatter plot. Notably, data point CMC-VP-10660 falls outside the morphospace for the defined colour categories, whereas data point 10662 sits within the morphospace of brown, grey and black colour categories. The authors’ interpretation of brown-toned integument is thus not supported by the data. Furthermore, the authors claim that sample VP-20858 preserves melanosomes with a distinct morphology to those in VP-10660. The authors use this observation to claim that sauropods had the potential to display complex epidermal colour patterns. Unfortunately, the VP-20858 data are not plotted in the scatter plot and are not reported in the supplementary material, so this claim cannot be verified. There are further inaccuracies regarding the data plotting and reporting: (i) the polygonal fields defining the melanosome morphospace for each colour category are not drawn correctly in figure 5; (ii) the unit labels in the supplementary table are incorrect, e.g. diameter1 has a unit in ‘>micro’, the radius is reported as ‘>mm²’ and ‘?mm²’. The radius of melanosomes is not a parameter usually measured and/or reported in the literature.

Critically, the study lacks statistical analyses to support the claim that the diplodocid skin melanosomes are disc-shaped and similar to those of feathers, and different to melanosomes in the skin of other reptiles. Because of this, the authors cannot claim that (i) two distinct melanosome morphotypes are present, and (ii) the specimens show potential colour pattern preservation. Statistical analysis and future use of the published data would be meaningless, as melanosomes are not fully exposed on the surface.

Finally, the quality of SEM and energy dispersive X-ray spectroscopy (EDS) analyses is not optimal for the interpretation of the structure and elemental chemistry of the samples. This is due to the low sample number and lack of appropriate preparation. The SEM–EDS data provided by Gallagher *et al.* [1] are based only on fractured, non-polished surfaces, which limits the visualization of discrete biological structures. Moreover, the SEM micrographs show charging artefacts, which can affect the elemental maps by highlighting features that are not real, compromising the interpretation of the elemental chemistry. The EDS maps in figure 4 are of a surface that is neither flat nor perpendicular to the beam, and thus the elemental maps include topographic artefacts. The reconstruction of the epidermal skin is thus not based on the data provided.

The work as presented does not meet the standard of current work in the field. Future efforts to reconstruct the colour of skin in sauropod dinosaurs will require new evidence of a link between integumentary melanosome characteristics and coloration and/or the recovery of evidence of pigment molecules and/or pigment cells in fossils.

## Data Availability

This article has no additional data.
